# Novel insights into the diversity of halophilic microorganisms and their functioning in hypersaline ecosystems

**DOI:** 10.1038/s44185-024-00050-w

**Published:** 2024-08-02

**Authors:** Aharon Oren

**Affiliations:** Department of Plant and Environmental Sciences, The Institute of Life Sciences, The Hebrew University of Jerusalem, Edmond J. Safra Campus, Jerusalem, 9190401 Israel

**Keywords:** Microbiology, Biogeochemistry

## Abstract

Our understanding of the microbial diversity inhabiting hypersaline environments, here defined as containing >100–150 g/L salts, has greatly increased in the past five years. Halophiles are found in each of the three domains of life. Many novel types have been cultivated, and metagenomics and other cultivation-independent approaches have revealed the existence of many previously unrecognized lineages. Syntrophic interactions between different phylogenetic lineages have been discovered, such as the symbiosis between members of the archaeal class *Halobacteria* and the ‘*Candidatus* Nanohalarchaeota’. Metagenomics techniques also have shed light on the biogeography of halophiles, especially of the genera *Salinibacter* (*Bacteria*) and *Haloquadratum* and *Halorubrum* (*Archaea*). Exploration of the microbiome of hypersaline lakes led to the discovery of novel types of metabolism previously unknown to occur at high salt concentrations. Studies of environments with high concentrations of chaotropic ions such as magnesium, calcium, and lithium have refined our understanding of the limits of life.

But the miry places thereof and the marishes thereof shall not be healed; they shall be given to salt

## Introduction

The above quotation (Ezekiel 47: 11) refers to the Dead Sea, the lake where I started exploring microbial diversity in hypersaline environments forty-five years ago. In spite of the extreme conditions that prevent most other life forms to thrive there, the Dead Sea and other hypersaline ecosystems are inhabited by a surprising diversity of microorganisms that are ‘given to salt’, and are adapted to live at salinities up to saturation, often in the presence of high concentrations of ions more toxic than Na^+^ and Cl^-^ that dominate thalassohaline (seawater-derived) hypersaline environments, high and low temperatures, high and low pH values, and more.

Here I review new insights, mainly obtained during the past five years, into the phylogenetic and metabolic diversity of halophilic microorganisms, operationally defined as organisms, cultivated as well as yet-uncultivated, growing at >100–150 g/L dissolved salts (Table 1), and their function in hypersaline ecosystems worldwide.

**Table 1:** Examples of genera of *Archaea*, *Bacteria*, and *Eukarya* that contain halophilic representatives, able to grow at salt concentrations >100–150 g/L, and their taxonomic affiliation

The list is not exhaustive. Names of taxa of *Archaea* and *Bacteria* that were not validly published under the provisions of the ICNP as of 15 April 2024 are printed in blue font. Prokaryotic nomenclature used is based in part on refs. ^90–92^ and on information derived from the List of names of Prokaryotes with Standing in Nomenclature (LPSN, https://www.bacterio.net or https://lpsn.dsmz.de). Names under the provisions of the ‘SeqCode’ – the Nomenclatural Code for Prokaryotes Described from Sequence Data are printed in red font. For members of the *Eukarya*, the taxonomy as given in Catalog of Life (https://www.catalogueoflife.org/) was followed.

## Halophiles in the domain *Archaea*

The best known group of extreme halophiles is represented by the archaeal phylum *Halobacteriota* (kingdom *Methanobacteriati*). In the archaeal domain we further find halophiles in the phylum *Methanobacteriota* (kingdom *Methanobacteriati*) and in the ‘*Candidatus’* phylum Nanohalarchaeota (kingdom *Nanobdellati*). The number of taxa recognized in the class *Halobacteria*, the halophiles par excellence, has greatly increased in recent years. As of December 2023, it encompassed nine families, 82 genera, and 357 species with validly published names^1^, an increase of about 50% since the census of May 2017 with six families, 57 genera, and 233 species^2^. Many of the earlier recognized taxa were reclassified following in-depth phylogenomic analyses based on concatenated conserved single-copy marker proteins. The number of orders in the class was reduced to two: *Halobacteriales* and *Halorutilales*; the earlier described orders *Haloferacales* and *Natrialbales* were unified with the *Halobacteriales*^3^. The recently described *Halorutilus salinus*, abundantly found in intermediate-to-high salinity ecosystems worldwide, is as yet the only representative of the *Halorutilales* order^4^.

New insights on the mode of osmotic adaptation of some members of the *Halobacteria* were obtained from the study of isolates of the genus *Halomicroarcula* (family *Haloarculaceae*). Members of the group typically use the ‘salt-in’ strategy by accumulating KCl^5^. However, *Halomicroarcula* strains from hypersaline soils from the Odiel saltmarshes, Spain, encode complete pathways for the biosynthesis of the osmotic solutes trehalose and glycine betaine^6^. Biosynthesis of organic compatible solutes may make the organisms more versatile and enable them to grow also under intermediate to low salinities. De novo biosynthesis of glycine betaine via the choline oxidation pathway was identified also in few additional members of the *Halobacteria*^7^.

The flat, square *Haloquadratum walsbyi* (family *Haloferacaceae*) is a prominent member of the microbial community of many salt lakes and saltern crystallizer ponds. Experiments in which the salinity of a Spanish model saltern pond was rapidly reduced from 340 to 120 g/L, leading to massive lysis of archaeal cells, showed presence of two ecotypes with different salt concentration preferences. The osmotic shock led to a temporary increase in the abundance of the originally less abundant ecotype that carried special genes related to solute transport (e.g., an ABC-type transport system of amino acids and other small organic compounds) and gene regulation^8^. The pangenome of *H. walsbyi* from a single saltern site is comparable to that of *Escherichia coli* collected from disparate ecosystems. While extensive intra-population gene diversity is found within a single site, only a small minority of these genes appears to be functionally important during environmental perturbations^9^.

A recent addition to the list of species of the family *Halobacteriaceae* is *Actinarchaeum halophilum*, isolated from a salt marsh in China. It displays a life cycle resembling that of *Streptomyces* (*Bacteria*, *Actinomycetota*), with cellular differentiation into mycelia and spores^10^.

Attempts were recently made to reconstruct the evolutionary history of the *Halobacteria* class and the other halophiles within the domain *Archaea*. The pangenome of the *Halobacteria* showed a core component of 300 genes, including genes for replication, transcription, translation and repair, and a variable component including a major portion involved in environmental information processing. Occurrence of horizontal gene transfer during the evolution of the *Halobacteria* was indicated by a high percentage of derived genes and presence of transformation and conjugation genes. The derived genes may have enabled the members of the group to colonize new environments and adapt to the new conditions^11^.

Another group of halophilic archaea is the ‘*Candidatus*’ phylum Nanohalarchaeota (kingdom *Nanobdellati*)^1^. None of its members have yet been grown in pure culture. The group was first recognized by metagenomic assembly of DNA libraries from surface water of the hypersaline Lake Tyrrell, Australia. Visualization using lineage-specific probes showed very small cells ~0.6 μm in diameter^12^. Members of this linage are abundant in hypersaline environments worldwide. A strain designated ‘*Candidatus* Nanohalarchaeum antarcticum’ was grown in co-culture with *Halorubrum lacusprofundi* (*Halobacteria*), showing that it is not free living but requires presence of a host. Metagenome-assembled genomes (MAGs) of members of the group code for unusually large proteins predicted to function in attachment to hosts. Genes for key biosynthetic pathways such as lipid synthesis are missing, showing that the nanohalarchaeota have evolved as symbionts^13^. A similar association was shown between ‘*Candidatus* Nanohalobium constans’ and a chitin-degrading *Halomicrobium* strain in a saltern crystallizer pond in Sicily, Italy. ‘*Candidatus* Nanohalobium’ can hydrolyze starch and glycogen, substrates that are not used by the *Halomicrobium*. Presence of the ectosymbiont thus increases the host’s metabolic capacity^14^. Two other extremely halophilic symbiotic nanohalarchaeota (‘*Candidatus* Nanohalococcus occultus’ and ‘*Candidatus* Nanohalovita haloferacivicina’) were grown in a xylose-degrading binary culture with *Haloferax lucentense* (*Halobacteriota*)^15^.

Another novel type of archaeal halophiles, affiliated with the phylum *Thermoplasmatota* (kingdom *Methanobacteriati*) and abundant in the sediment of a Chinese soda-saline lake, is the order ‘*Candidatus* Halarchaeoplasmatates with ‘*Candidatus*’ genera Halarchaeoplasma, Haladaptatiplasma, Saliniplasma, and Natronoplasma, all characterized from MAGs. Based on the isoelectric point profiles of predicted proteomes, the group probably uses the energetically favorable ‘salt-in’ strategy. The genomes code for degradation of carbohydrate and organic acids, as well as utilization of carbon monoxide and hydrogen for energy generation^16^.

Much has been speculated about the evolutionary origins of the different groups of archaeal halophiles, including the ‘*Candidatus* Nanohalarchaeota (*Nanobdellati*) and the *Methanonatronarchaeia*, a class of anaerobic methylotrophic methanogens (phylum *Methanobacteriota*) that includes neutrophilic and alkaliphilic members. The group is currently represented by single cultivated species, the neutrophilic (neutralophlic) *Methanonatronarchaeum thermophilum* from a hypersaline lake of the Kulunda Steppe, Altai, Russia^17^. Based on analysis of the highly conserved ATP synthase subunits, the ‘*Candidatus* Nanohalarchaeota’ are affiliated with the *Halobacteriota*^18^. Analysis of MAGs from hypersaline aquatic systems of the Danakil Depression (Ethiopia), two novel uncultivated lineages of halophilies were recognized, designated ‘Afararchaeaceae’ and ‘Asbonarchaeaceae’, which break the long branches at the base of the *Halobacteriota* and the ‘*Candidatus* Nanohalarchaeota’, respectively. Analysis of their sequences suggested that during the evolution of the Archaea, at least four independent adaptations to extreme halophily occurred. Gene duplication and horizontal gene transfer may have played an important role, e.g., by spreading key genes such as those encoding potassium transporters across extremely halophilic lineages^19^. Analysis of MAGs from soda-saline lakes on the Ordos Plateau (Inner Mongolia, China), representing a novel class designated ‘*Candidatus* Ordosarchaeia’, shed further light on the evolution of the archaeal halophiles. The group, inferred to have an aerobic chemoheterotrophic metabolism but containing remnants of the gene sequences of metabolism of methylated amines and coenzyme M biosynthesis, is positioned between the *Methanonatronarchaeia* and the *Halobacteriota* lineages. The *Methanonatronarchaeaa*, '*Candidatus* Ordosarchaeia and the *Halobacteriota* may share a common ancestor^20^.

## Halophiles in the domain *Bacteria*

The largest (>160 species) and best-known group of moderately halophilic bacteria with high salt tolerance is the family *Halomonadaceae* (kingdom *Pseudomonadati*, phylum *Pseudomonadota*). In-depth genomic analyses recently resulted in the division of the genus *Halomonas* into seven separate genera and the reclassification of seven *Halomonas* species in the genus *Modicisalibacter*^21^.

While *Halomonas* and relatives use organic osmotic solutes such as ectoine and glycine betaine for osmotic stabilization, *Salinibacter* (kingdom *Pseudomonadati*, phylum *Rhodothermota*) uses the ‘salt-in’ strategy known from the archaeal *Halobacteriota*. *Salinibacter ruber* is a major component of hypersaline aquatic ecosystems worldwide, and has become a popular model for evolutionary and ecological studies. As for the archaeon *Haloquadratum walsbyi*, the pangenome of *Salinibacter ruber* from a single saltern site is comparable to that of *E. coli* collected from many different ecosystems^9^.

Genomic analysis of eight *Salinibacter ruber* strains isolated from two Mediterranean salterns at two different time points showed contrasting evolutionary patterns in the core and accessory genomes. Extensive homologous recombination was found in the core genome, resulting in limited sequence variation within population clusters. Horizontal gene transfer was extensively encountered in the accessory genome. Restriction and modification or CRISPR-Cas systems modulated both modes of genetic exchange^22^. To define the concept of a bacterial ‘strain’ and to estimate how many strains are found in a natural population, the genomes of 138 *Salinibacter ruber* isolates from two solar salterns were compared with metagenomes from the same samples. A bimodal distribution was found of the genome-aggregate Average Nucleotide Identity (ANI) values among these isolates, with four-fold lower occurrence of values between 99.2% and 99.8% relative to values > 99.8% or <99.2%. Using an ANI value of >99.99% to define genomovars and >99.0% to define strains, it was estimated that the 138 isolates represented about 80% of the *S. ruber* strains in the population, and that 5500 to 11,000 genomovars were present in a single saltern pond, most of them being rare^23^.

The genus *Salinibacter* currently (April 2024) contains five species with names validly published under the rules of the International Code of Nomenclature of Prokaryotes (ICNP). The latest addition is *Salinibacter grassmerensis* from a lagoon in the northwestern South Island, New Zealand, close to the Cook Strait. Analysis of genomes of thousands of *Salinibacter* isolates and MAGs from sites worldwide showed evidence for at least three more species^24^.

## Uncultivated prokaryotic lineages revealed by cultivation-independent approaches

Cultivation-independent methods were used in numerous recent studies to characterize hypersaline ecosystems. Many sequences belonging to novel phylogenetic lineages were discovered. The prokaryotic communities from hypersaline sapropels in the Transylvanian Basin, Romania (72–112 g NaCl/kg) yielded sequences affiliated with 59 phyla or ‘*Candidatus*’ phyla. *Pseudomonadota*, *Bacteroidota* and *Chloroflexota* were most abundant. Members of 32 candidate divisions and other undocumented prokaryotic lineages were found, showing that hypersaline sapropels accommodate extremely diverse ecosystems^25^. Another interesting environment is the hypersaline (up to 250 g/L during low tide) microbial mats at Shark Bay, Western Australia. MAGs of microbial ‘dark matter’ included 42 ‘*Candidatus*’ phyla including ‘Zixibacteriota’, ‘Parcubacteriota’, ‘Asgardarchaeota’, ‘Bathyarchaeota’ and members of the *Nanobdellati* kingdom^26^.

## Halophiles in the domain Eukarya

Hypersaline ecosystems are generally dominated by prokaryotes, but eukaryotic halophiles also exist. The best knowns are the unicellular green algal genus *Dunaliella*, which is the main primary producer in most high-salt aquatic systems, the fungi *Hortaea werneckii* (Ascomycota) and *Wallemia ichthyophaga* (Basidiomycota), and the brine shrimp *Artemia*.

The genome of *Dunaliella salina* strain CCAP 19/18 was recently sequenced. The genome is large (343.7 Mbp), 53% is contained in introns, and it has 16,667 loci coding for 18,801 protein-coding transcripts. Genome-based capabilities for the metabolism of glycerol, the osmotic solute produced by *Dunaliella*, were elucidated, as well as the pathways leading to the formation of carotenoids (exploited commercially using this alga). Carotenoid biosynthesis pathways of *D*. *salina* include prokaryotic-type phytoene desaturases, thus eukaryotic and prokaryotic elements are both present^27^.

Halophilic fungi are not only found in hypersaline lakes, but also in glacial ice, as freezing causes the displacement of solutes from expanding ice crystals into the liquid water between the crystals. Such extremophilic or extremotolerant fungi possess the necessary mechanisms to balance cellular osmotic pressure and ion concentration, stabilize cell membranes, and neutralize intracellular oxidative stress^28^. They are typically slow growing, as large amounts of energy are diverted into cellular mechanisms necessary for survival under hostile conditions. The polymorphism and meristematic growth of such fungi may be an adaptation to life under extreme conditions^29^.

The extremely halotolerant fungus *Hortaea werneckii* can form an association with *Dunaliella atacamensis*, an alga discovered in a cave in the Atacama Desert (Chile), and with *D. salina*, which is commonly found in salt lakes and saltern brines. In such ‘borderline lichens’, *D. atacamensis* forms small colonies that include *H. werneckii* cells. No mutual advantages were yet demonstrated for the partners in these associations^30^.

Different types of protists can also grow at salt concentrations approaching saturation. Analysis of 18S rRNA gene libraries of brine from a solar saltern in Taean County on the west coast of the Republic of Korea yielded a great diversity of Alveolata, Stramenopila, Archaeplastida, and Opisthokonta. The brine was found to harbor a large number of yet undescribed Amoebozoa, Cryptista, Haptista, Rhizaria, and Stramenopila^31^.

## Diversity of viruses in hypersaline environments

A study of the diversity of viruses and virus–host interactions in sediments of different salinities of Great Salt Lake (Utah), showed presence of haloviruses and members of families *Siphoviridae*, *Myoviridae*, and *Podoviridae*. Computational host predictions revealed a dominance of viruses that infect *Pseudomonadota*, *Actinomycetota*, and *Bacillota*. Auxiliary metabolic genes for photosynthesis (*psbA*), carbon fixation (*rbc*L, *cbb*L), formaldehyde assimilation (SHMT), and nitric oxide reduction (*Nor*Q) were identified in the viral genomes^32^. Genomes of lambda-like phages, phages of *Halomonas*, and 27 partial novel halophilic viral genomes were retrieved from high altitude thalassohaline environments in the Peruvian Andes^33^. Four new viruses infecting halophilic archaea were isolated from the hypersaline Lake Retba (Senegal). Three of these possess enveloped pleomorphic virions and were assigned to the *Pleolipoviridae*; the forth, designated HFTV1, has an icosahedral capsid and a long non-contractile tail, typical of the *Caudovirales*. It was isolated on a *Haloferax* strain, and could also infect *Halorubrum* sp^34^. An inventory of archaeal virus sequences in metatranscriptomes of Lake Tyrrell (Australia) and cultures seeded from four Antarctic lakes yielded 12 divergent RNA virus-like sequences affiliated with the *Artverviricota*, *Duplornaviricota*, *Kitrinoviricota*, *Negarnaviricota*, and *Pisuviricota*. However, no RNA viruses were detected using archaeal CRISPR spacers as a BLAST database. In addition, DNA viruses from the families *Pleolipoviridiae*, *Sphaerolipoviridae*, *Halspiviridae*, and the class *Caudoviricetes* were found^35^. Viral genomes were also retrieved from underground water (~230 g/L total salinity) that feeds hypersaline springs in the Añana Salt Valley (Spain)^36^. Hypersaline environments thus have a large potential for discovering novel diversity of haloviruses, which may mediate horizontal gene transfer by transduction and contribute to our understanding of the diversity and functional evolution of halophilic microbial communities^37^.

## Cultivation-independent characterization of the microbial communities in thalassohaline hypersaline environments

Most recent studies characterizing microbial diversity in hypersaline environments used cultivation-independent methods, from small-subunit rRNA libraries to high-throughput metagenomics. Below is a selection of studies performed in recent years at NaCl-dominated near-neutral sites on different continents.

Lake Ursu (Romania) is a stratified lake which below a depth of 4 m is anoxic, sulfidic, and hypersaline (>350 g/L dissolved salts; *a*_w_ (water activity) down to 0.762). The hypersaline stratum harbors a phylogenetically diverse population of heterotrophs belonging to yet uncultivated lineages of the *Candidatus* phyla ‘Acetothermota’, ‘Cloacimonadota’, ‘Neomarinimicrobiota’, ‘Omnitrophota’, and others groups^38^.

Surprisingly, the first microbial exploration of the Aral Sea, located between Kazakhstan and Uzbekistan, was only recently performed. In the past decades, this lake has changed from a moderately saline water body to a hypersaline lake. Archaeal 16S rRNA gene sequences were dominated by *Halobacteriales*, many representing a novel cluster in the *Haloferacaceae* family. Bacterial diversity was mainly represented by *Pseudomonadota*, *Actinomycetota*, and *Bacteroidota*^39^. A recent survey of the Western Aral Sea (220 g/L salts) showed dominance of *Haloferacaceae* and the bacterial genera *Spiribacter* and *Psychroflexus*. In the sediments, archaea were less abundant and were dominated by ‘*Candidatus* Woesearchaeota’. *Bacteria* were mainly represented by sulfate reducers of the phylum *Desulfobacterota* and the genera *Fusibacter*, *Halanaerobium*, *Guyparkeria*, *Marinobacter*, *Idiomarina*, and *Thiomicrospira*^40^.

Metagenomic analysis of brine from Lake Urmia (Iran) (~270 g/L salts) showed dominance of *Haloquadratum* and *Halonotius* (*Haloferacaceae*). *Salinibacter ruber* was the main representative of the *Bacteria*^41^.

The upper 30 cm of the surface salt crust of the Bonneville Salt Flats, a salt pan at the Utah-Nevada border, harbors a microbial community dominated by members of the *Halobacteria* and *Salinibacter*. Sequences of *Geitlerinema*, a cyanobacterium that can use sulfide as the electron donor for photosynthesis, were also found. From the gypsum sediment layer below the surface halite, 16S rRNA genes of *Thermoplasmatales*, ‘*Candidatus* Hadarchaeota’, *Nanobdellota*, ‘*Candidatus* Acetithermia’, *Bacteroidota*, members of the *Halanaerobiales*, and the genera *Desulfovermiculus* and *Rhodovibrio* were recovered^42^.

MAGs related to the elusive ‘*Candidatus* Patescibacteria’ were detected in relatively high abundance (4.5% of the sequences) in hypersaline brine sampled through a borehole in a coastal glacier in Northern Victoria Land, Antarctica. Archaeal sequences were dominated by *Methanoculleus* (*Methanomicrobia*). More than a quarter of the fungal sequences could not be assigned to known taxa^43^.

Deep-sea hypersaline anoxic basins on the bottom of the Mediterranean Sea, the Red Sea, and the Gulf of Mexico are among the most extreme ecosystems. Recent comprehensive reviews document the prokaryotic diversity encountered^44,45^. Some contain NaCl-dominated brines. An example is the Orca Basin, the largest brine basin in the Gulf of Mexico. The 16S rRNA gene clone libraries from its hypersaline sediments and the overlying brine (255–267 g/L salts) were dominated by the uncultivated halophilic KB1 lineage affiliated with the ‘*Candidatus* Acetothermota’ phylum, *Deltaproteobacteria* related to sulfate-reducing genera, and *Bacteroidota*. Archaea were dominated by *Methanohalophilus* and the ammonia-oxidizing Marine Group I (kingdom *Thermoproteati*)^46^. Metagenomic libraries at a 10-cm-scale resolution along the 1-m salinity gradient between the overlaying seawater and the 3.5-times as saline Suakin Deep located at 2770 m in the central Red Sea revealed fine-scale community structuring and vertical succession of metabolic groups^47^. Different types of mobile antibiotics resistance genes were detected in DNA isolated from different Red Sea brine pools^48^.

## Cultivation-independent characterization of the microbial communities in athalassohaline hypersaline environments

Some deep-sea hypersaline brines are dominated by ions other than Na^+^ and Cl^-^ (‘athalassohaline brines’). Thus, the Kryos, Discovery, and Hephaestus basins located in the Eastern Mediterranean Sea contain near-saturated solutions of MgCl_2_. The *a*_w_ of such brines is much lower than that of saturated NaCl solutions. Moreover, in contrast to the stabilizing (‘kosmotropic’) effect of NaCl, MgCl_2_ has a destabilizing (‘chaotropic’) effect on biomolecules, making such environments much more extreme for life than NaCl brines^49^. An investigation of the microbial community structure and activities across the interface of the brine and the overlying seawater of the Kyros Basin suggested the occurrence of sulfate reduction in the brine (*a*_w_ ~ 0.4), probably due to activity of *Desulfovermiculus* and *Desulfobacula* (*Deltaproteobacteria*). However, whether indeed life is possible at such a low *a*_w_ value needs further confirmation. In the lower part of the interface, elevated rates of methane oxidation were measured under micro-oxic conditions^50^. To assess the upper limit of MgCl_2_ concentration for life, signatures of life were analyzed in the gradient from seawater (0.05 M) to the deep brine (5.05 M) of Discovery Basin. Growth of microbes collected from different parts of the interface was inhibited at >1.26 M MgCl_2_. DNA and rRNA of sulfate reducers and methanogens were detected along the entire MgCl_2_ gradient, but the much more labile mRNA, an indicator of active microbes, was recovered only up to 2.3 M. In the absence of kosmotropic solutes, this may be the upper concentration for life^51^. To further explore the limits of life in MgCl_2_-rich brines, 16S rRNA genes and microbial activities were quantified at a salt harvesting facility in California in a series of ponds from NaCl brines to highly chaotropic MgCl_2_ brines. Exogenous genetic material entering the chaotropic brines is preserved there, resulting in an unexpected increase in apparent microbial diversity in MgCl_2_-saturated brines^52^. Magnesium sulfate appears to be less toxic than magnesium chloride: isolates of *Halomonas* and *Marinococcus* from the MgSO_4_-rich Basque Lake (British Columbia) and Hot Lake (Washington) grew well in saturated MgSO_4_ medium (67%) at 25 °C^53^. On the other hand, a study of the response of *Bacillus subtilis* to different mixtures of Na^+^, Mg^2+^, Ca^2+^, Cl^-^, SO_4_^2-^, and ClO_4_^-^ showed that chloride salts allow growth at lower *a*_w_ than sulfate salts. Despite the theoretically counteracting disordering (chaotropic) effects of perchlorates and ordering (kosmotropic) effects of sulfates, combination of their Na^+^ or Mg^2+^ salts additively narrowed the window for growth. Thus, the limits of life in mixed ion solutions may be specific to the salts and the organisms in question, rather than to *a*_w_, ionic strength or chaotropicity^54^. Nanoscale secondary ion mass spectrometry was used to assess anabolic activity in nearly 6000 individual cells from solar saltern sites with *a*_w_ ranging from 0.982 (seawater) to 0.409 (MgCl_2_-dominated brine). Activity, as measured by net carbon and/or nitrogen assimilation from ^13^C^15^N-amino acids, ^15^N-ammonium, ^15^N-nitrate, ^13^C-bicarbonate, and ^13^C-glucose, decreased exponentially with *a*_w_. No microbial activity was detected at *a*_w_ 0.409, despite the presence of cell-like structures. The *a*_w_ limit for detectable anabolic activity was estimated at 0.540^55^.

Another chaotropic salt is CaCl_2_. The bottom brines (150 g/L salts) and the sediment of the perennially ice-covered Lake Vanda (McMurdo Dry Valleys, Antarctica) support sulfate reduction and methanogenesis through the methylotrophic, acetoclastic, and hydrogenotrophic pathways. Sequencing of 16S rRNA gene libraries showed dominance of *Pseudomonadota*, *Bacillota* including abundant presence of the *Halanaerobiales*, and *Bacteroidota*. Surprisingly, sulfate-reducing *Deltaproteobacteria* were not observed. The majority of the archaeal sequences belonged to the *Thermoplasmata* class^56^.

Lithium chloride can also be a chaotropic stressor. Three large salars (salt flats), Salar de Atacama (Chile), Salar de Uyuni (Bolivia), and Salar del Hombre Muerto (Argentina), contain the largest lithium reserves on Earth. The Salar de Uyuni reaches saturation for NaCl, and contains high concentrations of MgCl_2_ and LiCl. Temperature and humidity fluctuations and exposure to high UV radiation increase the extremity of this environment. Small subunit rRNA gene libraries from four sampling stations (Na^+^ 3.5–4.7 M, Mg^2+^ 0.2–1.9 M, Li^+^ 0.04–0.18 M) yielded sequences of *Archaea* (classes *Halobacteria*, *Thermoplasmata* and ‘*Candidatus* Nanohalarchaeota’) and Bacteria (mainly *Bacteroidota* and *Pseudomonadota*)^57^. *Halonotius* and *Halorubrum* were the most abundant archaeal species, *Salinibacter* was the most common bacterial member of the community^58^. A cultivation-dependent study of the Salar del Hombre Muerto microbial community showed 30% of the 238 isolates to grow in solid medium proximally to a LiCl solution close to saturation (15 M). Most of these belonged to the genera *Bacillus*, *Micrococcus*, and *Brevibacterium*. Isolates of *Kocuria*, *Curtobacterium* and *Halomonas* tolerated 0.7–1.4 M LiCl^59^. Two *Bacillus* isolates from the Salar de Atacama (556 g/L total salts; 11.7 M LiCl) still grew in the presence of 1.44 M Li^+^, albeit at reduced rates^60^.

Arsenic is another toxic element found in high concentrations in some hypersaline ecosystems. Halophilic *Archaea* abound in a red biofilm in Diamante Lake in the Andean Puna, Argentina (270 g/L total salts, pH 9–11, 115–234 mg/L arsenic). A metagenomics survey revealed a high abundance of genes for anaerobic arsenate respiration (*arr*) and arsenite oxidation (*aio*). A number of arsenic-tolerant *Halorubrum* strains were isolated that harbored *aio* and *arr* genes; one of the isolates was able to oxidize As[III]^61^. Metagenomic analysis of the Salar de Ascotán, a high-altitude arsenic-rich salt flat in the Atacama Desert, Chile, showed predominance of *Pseudomonadota*, *Acidobacteriota*, and *Bacteroidota*, as well as *Archaea*. MAGs were retrieved of representatives of the ‘*Candidatus* Patescibacteria’, *Pseudomonadota*, and two novel archaeal lineages of *Halobacteria* and *Thermoplasmata*. Genes for resistance to arsenic and metals such as copper, cadmium, cobalt, nickel, and zinc were widely found^62^.

One of the most challenging hypersaline environments for life is the Dallol volcano and its associated hydrothermal field located in the northern Danakil Depression, Ethiopia. Several small lakes with 340–410 g/L total salts have NaCl-based brines with high chaotropicity due to high Mg^2+^ and Ca^2+^ concentrations, and pH values below 5^63–66^. If life was detected at all, it was dominated by ultra-small archaea^61^. 16S rRNA gene amplicon sequences of *Halobacteriota* and ‘*Candidatus* Nanohalarchaeota’ were reported in the earlier studies. Microscopic examination of natural samples and enrichment cultures suggested presence of different types of active cells, and scanning electron microscopy showed small (0.2–0.3 μm) cells associated with somewhat larger (0.6–1 μm) cells^65^. However, no life was detected in the hyperacidic (pH ~ 0), hypersaline (~350 g/L) and sometimes hot (up to 108 °C) ponds of the Dallol dome. It is plausible that aerosols had transported halophilic archaea likely originating from neighboring hypersaline ecosystems to the lakes, in addition to bacteria typically found in soil and dust. Moreover, DNA fluorescent probes and dyes may unspecifically bind to mineral precipitates in the Dallol brines. Cells were found to be rapidly degraded upon contact with the chaotropic hyperacidic brine. Therefore, the earlier positive results of life detection approaches need to be reevaluated^66^.

## Biogeography of halophilic microorganisms

Hypersaline environments are found on all continents, but are often far removed from each other. Therefore, halophilic microorganisms are excellent model organisms to explore biogeographic patterns. It is interesting to note that on the same pages on which Lourens Baas Becking disclosed his “Alles is overal, maar het milieu selcteert” (Everything is everywhere, but, the environment selects) hypothesis, he gave examples from the world of the halophilic microorganisms^67^.

One of the possible mechanisms by which halophilic prokaryotes may be dispersed, is within the nostril glands or on the feathers of birds, as documented for shearwaters, flamingoes, and pelicans^68–70^. A study of *Halobacteria* associated with halite crystals collected from coastal salterns of Western Europe, the Mediterranean, and East Africa yielded little support for the existence of biogeographical regions for this group of *Archaea*, although some taxa showed biogeographical patterns^71^. Analysis of fifty hypersaline brine and sediment samples from Europe, Spanish-Atlantic and South America for biogeographical patterns in the archaeal and bacterial community structure revealed regionally distinct taxa compositions at the species level, but less so at the level of genus and higher taxa^72^.

The prominence of members of the genus *Halorubrum* (*Halobacteria*, a genus found in hypersaline environments worldwide) in hypersaline cold Antarctic lakes is supported by the existence of a low-temperature adapted clade. Six isolates from polar and deep earth environments were distinguished from other *Halorubrum* strains by a lower G + C content and different amino acid composition. The group was characterized by increased flexibility of the proteins encoded and a denser genome packing relative to the reference group^73^. The Antarctic *Halorubrum lacusprofundi* contains more than one replicon. Assessment of genomic variation between strains of *H. lacusprofundi* isolated from different Antarctic locations and metagenomes from six hypersaline Antarctic lakes showed that the sequence of the largest replicon of each strain was highly conserved, while the two smaller replicons were highly variable. Differences were also found in susceptibility to a halovirus. Metagenome data demonstrated that specific haloarchaeal species are endemic to Antarctica, and show biogeographical variation consistent with environment and distance effects^74^. The Antarctic lakes can therefore be used as model systems to test Baas Becking’s “Everything is everywhere, but, the environment selects” hypothesis. Endemism may be due to their environmental specificity, or to geographical isolation.

## Novel insights into the metabolic diversity in hypersaline ecosystems

Not all types of metabolism known from low-salt ecosystems function at the highest salinities. Notable examples are autotrophic nitrification and methanogenesis from H_2_ and CO_2_ and from acetate. The upper salt concentration at which dissimilatory processes can occur can be understood based on bioenergetic considerations. The amount of energy generated during dissimilatory metabolism and the mode of osmotic adaptation used (energetically expensive de novo biosynthesis of organic solutes or the less expensive ‘salt-in’ strategy based on KCl accumulation) can explain nearly all observations^75,76^.

In recent years, our understanding of the sulfur cycle in neutral and alkaline hypersaline ecosystems has greatly increased. Anaerobic elemental sulfur-respiring archaea of the class *Halobacteria*, a group that typically consists of aerobic heterotrophs, are abundant in sediments from hypersaline soda lakes^77^. Isolates include *Natronolimnobius sulfurireducens* from soda lakes at different locations and *Halalkaliarchaeum desulfuricum* from Searles Lake (California). Formate, hydrogen, small fatty acids and peptone can serve as electron donors and sulfur or dimethylsulfoxide as electron acceptors^78^. *Natranaeroarchaeum sulfidigenes* from soda lakes in southwestern Siberia is a facultative anaerobe that in the absence of oxygen uses α-d-glucans (amylopectin, amylose, glycogen), sugars, or glycerol as electron donors with elemental sulfur or thiosulfate as electron acceptors. Under microaerophilic conditions, oxygen can serve as the electron acceptor^79,80^. Another electron donor that may drive sulfide respiration in hypersaline environments is carbon monoxide. In an anaerobic methanogenic enrichment of sediment from hypersaline soda lakes, CO was oxidized using the Wood-Ljungdahl pathway by a novel bacterium named *Natranaerofaba carboxydovora* (*Natranaerobiales*, *Bacillota*). This moderate thermophilic, halophilic, and alkaliphilic acetogen uses CO, formate, pyruvate, and lactate as electron donors and thiosulfate, nitrate, and fumarate as electron acceptors^81^. Aerobic oxidation of CO by extremely halophilic members of the *Archaea* was first documented in a *Halorubrum* strain isolated from the Bonneville Salt Flats (Utah)^82^. Strains of *Halanaeroarchaeum* and *Halalkaliarchaeum* were isolated from hypersaline sulfidic salt lake and soda lake sediments, respectively, with CO as electron donor and elemental sulfur as electron acceptor^83^.

Metagenomic analysis of anoxic cold (~ –5 ^°^C) hypersaline (~240 g/L) sulfate-rich brine from Lost Hammer Spring, located in the Canadian High Arctic, showed prominent presence of lithoautotrophic sulfide-oxidizing *Gammaproteobacteria*. Evidence was also found for the presence of sulfate reducers and anaerobic methane-oxidizing archaea^84^.

Anaerobic enrichment cultures were set up at 4.4 M NaCl, using hydrogen as the electron donor and inoculated with material from salt caverns at different locations in Europe to be used as underground gas storage sites. In the absence of other potential electron donors, the acetogenic *Acetohalobium* (*Halanaerobiales*, *Bacillota*) dominated; sulfate reduction was most likely associated with a member of the *Desulfonatronovibrionales* (*Deltaproteobacteria*)^85^.

In summary, the study of metabolic diversity in hypersaline environments provides insights into the possibilities and limitations of life under the most extreme conditions, and has recently led to the recognition of a number of unusual types of metabolism never documented before in high-salt environments.

## Final comments

Application of state-of-the art methods of metagenomics and other cultivation-independent approaches to study microbial diversity has led to the recognition of many previously unknown types of organisms belonging to different phylogenetic lineages, and inhabiting hypersaline environments worldwide. While much information can be deduced from analysis of MAGs, the ultimate goal should be a study of all those novel types in culture. ‘Culturomics’ methods can be useful, as shown in the case of the isolation of the archaeon *Halorutilus*^4^. Others were discovered serendipitously, for example the intriguing *Actinarchaeum halophilum* that grew during a study aimed at the isolation of halophilic *Actinomycetota* from a Chinese salt lake^9^. Isolation in pure culture may not always be possible when different types of halophiles depend on each other because of mutualistic relationships. Examples were found during the study of ‘*Candidatus* Nanohalarchaeota’^13–15^, the lichen-like association of *Dunaliella atacamensis* and the halophilic fungus *Hortaea werneckii*^29^, and the metabolic cross-feeding discovered between a *Halorubrum* strain and a *Marinococcus* from the Cuatro Cienegas Basin (Mexico), growing together at 250 g/L salt^86^.

Many hypersaline environments that were explored in the past for their microbial diversity are rapidly changing. Thus, Great Salt Lake (Utah) reached its lowest level in recorded history in 2022; the water level of Lake Urmia (Iran) dropped more than 7 meters and the lake lost ~90% of its area since 1995^87^. The salinity of the brines increased accordingly, making these lakes more extreme as a habitat for microorganisms.

The same is true for the Dead Sea, the lake I started exploring nearly half a century ago. To my knowledge the last published attempt to characterize the microbial community in its waters was based on a surface water sample collected in June 2015. No information was given about the community density, but based on my investigation of the lake, microorganisms must have been very rare. The brine, containing 340 g/L total salts, was dominated by chaotropic cations (1.90 M Mg^2+^ and 0.59 M Ca^2+^ and only 1.49 M Na^+^ and 0.11 M K^+^). Analysis of 16S rRNA gene libraries showed 45% archaeal sequences, especially affiliated with *Halorhabdus* (a genus detected also in an earlier census made in 2007^88^) and *Natronomonas*; the remainder were mainly bacterial sequences of *Pseudomonadota* and *Bacillota* with a minor contribution of *Bacteroidota*, *Actinomycetota* and *Cyanobacteriota*^89^. Between 2007 and 2015, the water level dropped by about nine meters, and at the time of writing, the level is more than nine meters lower than in 2015. Precipitation of halite to the lake bottom and a concomitant increase in the relative concentrations of chaotropic ions make the places that are “given to salt” an ever more hostile environment for life.

### Note

Where relevant, names of phyla and kingdoms as given in the original publications were corrected to validly published names under the rules of the ICNP^90,91^. If necessary, names of *Candidatus* phyla were corrected as proposed^92^.

## References

[1] Cui, H.-L. et al. Proposed minimal standards for description of new taxa of the class *Halobacteria*. *Int. J. Syst. Evol. Microbiol.* **74**, 006290 (2024). 38456846 10.1099/ijsem.0.006290 PMC10999741

[2] Arahal, D. R., Oren, A. & Ventosa, A. International committee on systematics of prokaryotes subcommittee on the taxonomy of *Halobacteria* and subcommittee on the taxonomy of *Halomonadaceae*. Minutes of the joint open meeting, 11 July 2017, Valencia, Spain. *Int. J. Syst. Evol. Microbiol.* **67**, 4279–4283 (2017). 28857024 10.1099/ijsem.0.002296 PMC5845653

[3] Cui, C., Han, D., Hou, J. & Cui, H.-L. Genome-based classification of the class *Halobacteria* and description of *Haladaptataceae* fam. nov. and *Halorubellaceae* fam. nov. *Int. J. Syst. Evol. Microbiol* **73**, 005984 (2023). 10.1099/ijsem.0.005984 37486319

[4] Durán-Viseras, A. et al. Discovery of the streamlined haloarchaeon *Halorutilus salinus*, comprising a new order widespread in hypersaline environments across the world. *mSystems* **8**, e0119822 (2023). 36943059 10.1128/msystems.01198-22 PMC10134839

[5] Gunde-Cimerman, N., Plemenitaš, A. & Oren, A. Strategies of adaptation of microorganisms of the three domains of life to high salt concentrations. *FEMS Microbiol. Rev.* **42**, 353–375 (2018). 29529204 10.1093/femsre/fuy009

[6] Durán-Viseras, A., Sánchez-Porro, C. & Ventosa, A. genomic insights into new species of the genus *Halomicroarcula* reveals potential for new osmoadaptative strategies in halophilic archaea. *Front. Microbiol.* **12**, 751746 (2021). 34803972 10.3389/fmicb.2021.751746 PMC8600319

[7] Yang, N., Ding, R. & Liu, J. Synthesizing glycine betaine via choline oxidation pathway as an osmoprotectant strategy in *Haloferacales*. *Gene* **847**, 146886 (2022). 36108788 10.1016/j.gene.2022.146886

[8] Viver, T. et al. Distinct ecotypes within a natural haloarchaeal population enable adaptation to changing environmental conditions without causing population sweeps. *ISME J.* **15**, 1178–1191 (2021). 33342997 10.1038/s41396-020-00842-5 PMC8182817

[9] Konstantinidis, K. T., Viver, T., Conrad, R. E., Venter, S. N. & Rossello-Mora, R. Solar salterns as model systems to study the units of bacterial diversity that matter for ecosystem functioning. *Curr. Opin. Biotechnol.* **73**, 151–157 (2022). 34438234 10.1016/j.copbio.2021.07.028

[10] Tang, S. K. et al. Cellular differentiation into hyphae and spores in halophilic archaea. *Nat. Commun* **14**, 1827 (2023). 37005419 10.1038/s41467-023-37389-w PMC10067837

[11] Gaba, S., Kumari, A., Medema, M. & Kaushik, R. Pan-genome analysis and ancestral state reconstruction of class halobacteria: probability of a new super-order. *Sci. Rep.* **10**, 21205 (2020). 33273480 10.1038/s41598-020-77723-6 PMC7713125

[12] Narasingarao, P. et al. De novo metagenomic assembly reveals abundant novel major lineage of Archaea in hypersaline microbial communities. *ISME J.* **6**, 81–93 (2012). 21716304 10.1038/ismej.2011.78 PMC3246234

[13] Hamm, J. N. et al. Unexpected host dependency of Antarctic Nanohaloarchaeota. *Proc. Natl Acad. Sci. USA* **116**, 14661–14670 (2019). 31253704 10.1073/pnas.1905179116 PMC6642349

[14] La Cono, V. et al. Symbiosis between nanohaloarchaeon and haloarchaeon is based on utilization of different polysaccharides. *Proc. Natl Acad. Sci. USA* **117**, 20223–20234 (2020). 32759215 10.1073/pnas.2007232117 PMC7443923

[15] Reva, O. et al. Functional diversity of nanohaloarchaea within xylan-degrading consortia. *Front. Microbiol.* **14**, 1182464 (2023). 37323909 10.3389/fmicb.2023.1182464 PMC10266531

[16] Zhou, H. et al. Metagenomic insights into the environmental adaptation and metabolism of *Candidatus* Haloplasmatales, one archaeal order thriving in saline lakes. *Environ. Microbiol.* **24**, 2239–2258 (2022). 35048500 10.1111/1462-2920.15899

[17] Sorokin, D. Y. et al. *Methanonatronarchaeum thermophilum* gen. nov., sp. nov. and ‘*Candidatus* Methanohalarchaeum thermophilum’, extremely halo(natrono)philic methyl-reducing methanogens from hypersaline lakes comprising a new euryarchaeal class *Methanonatronarchaeia* classis nov. *Int. J. Syst. Evol. Microbiol.* **68**, 2199–2208 (2018). 29781801 10.1099/ijsem.0.002810 PMC6978985

[18] Feng, Y. et al. The evolutionary origins of extreme halophilic archaeal lineages. *Genome Biol. Evol.* **13**, evab166 (2021). 34255041 10.1093/gbe/evab166 PMC8350355

[19] Baker, B. A. et al. Expanded phylogeny of extremely halophilic archaea shows multiple independent adaptations to hypersaline environments. *Nat. Microbiol.* **9**, 964–975 (2024). 38519541 10.1038/s41564-024-01647-4

[20] Zhao, D. et al. Members of the class Candidatus Ordosarchaeia imply an alternative evolutionary scenario from methanogens to haloarchaea. *ISME J.* **18**, wrad033 (2024). 38366248 10.1093/ismejo/wrad033 PMC10873845

[21] de la Haba, R. R. et al. A long-awaited taxogenomic investigation of the family *Halomonadaceae*. *Front. Microbiol.* **14**, 1293707 (2023). 38045027 10.3389/fmicb.2023.1293707 PMC10690426

[22] González-Torres, P. & Gabaldón, T. Genome variation in the model halophilic bacterium *Salinibacter ruber*. *Front. Microbiol.* **9**, 1499 (2018). 30072959 10.3389/fmicb.2018.01499 PMC6060240

[23] Viver, T. et al. Towards estimating the number of strains thatmake up a natural bacterial population. *Nat. Commun.* **15**, 544 (2024). 38228587 10.1038/s41467-023-44622-z PMC10791622

[24] Viver, T. et al. Description of two cultivated and two uncultivated new *Salinibacter* species, one named following the rules of the bacteriological code: *Salinibacter grassmerensis* sp. nov.; and three named following the rules of the SeqCode: *Salinibacter pepae* sp. nov., *Salinibacter abyssi* sp. nov., and *Salinibacter pampae* sp. nov. *Syst. Appl. Microbiol.* **46**, 126416 (2023). 10.1016/j.syapm.2023.126416 36965279

[25] Andrei, A. Ş. et al. Hypersaline sapropels act as hotspots for microbial dark matter. *Sci. Rep.* **7**, 6150 (2017). 28733590 10.1038/s41598-017-06232-w PMC5522462

[26] Wong, H. L. et al. Microbial dark matter filling the niche in hypersaline microbial mats. *Microbiome* **8**, 135 (2020). 32938503 10.1186/s40168-020-00910-0 PMC7495880

[27] Polle, J. E. W. et al. Genomic adaptations of the green alga *Dunaliella**salina* to life under high salinity. *Algal Res*. **50**, 101990 (2020). 10.1016/j.algal.2020.101990

[28] Gostinčar, C. & Gunde-Cimerman, N. Understanding fungi in glacial and hypersaline environments. *Annu. Rev. Microbiol.* **77**, 89–109 (2023). 37001148 10.1146/annurev-micro-032521-020922

[29] Gostincar, C., Zalar, P. & Gunde-Cimerman, N. No need for speed: slow development of fungi in extreme environments. *Fungal Biol. Rev.* **39**, 1–14 (2022). 10.1016/j.fbr.2021.11.002

[30] Muggia, L. et al. The beauty and the yeast: can the microalgae *Dunaliella* form a borderline lichen with *Hortaea werneckii*? *Symbiosis* **82**, 123–131 (2020). 33536700 10.1007/s13199-020-00697-6 PMC7116670

[31] Lee, H. B. et al. The diversity patterns of rare to abundant microbial eukaryotes across a broad range of salinities in a solar saltern. *Microb. Ecol.* **84**, 1103–1121 (2022). 34779881 10.1007/s00248-021-01918-1 PMC9747883

[32] Bhattarai, B., Bhattacharjee, A. S., Coutinho, F. H. & Goel, R. K. Viruses and their interactions with bacteria and archaea of hypersaline Great Salt Lake. *Front. Microbiol.* **12**, 701414 (2021). 34650523 10.3389/fmicb.2021.701414 PMC8506154

[33] Castelán-Sánchez, H. G. et al. Intermediate-salinity systems at high altitudes in the Peruvian Andes unveil a high diversity and abundance of bacteria and viruses. *Genes* **10**, 891 (2019). 31694288 10.3390/genes10110891 PMC6895999

[34] Mizuno, C. M. et al. Novel haloarchaeal viruses from Lake Retba infecting *Haloferax* and *Halorubrum* species. *Environ. Microbiol.* **21**, 2129–2147 (2019). 30920125 10.1111/1462-2920.14604

[35] Le Lay, C. et al. Viral community composition of hypersaline lakes. *Virus Evol.* **9**, vead057 (2023). 37692898 10.1093/ve/vead057 PMC10492444

[36] Ramos-Barbero, M. D. et al. Ancient saltern metagenomics: tracking changes in microbes and their viruses from the underground to the surface. *Environ. Microbiol.* **23**, 3477–3498 (2021). 34110059 10.1111/1462-2920.15630

[37] Liu, Y. et al. Diversity, taxonomy, and evolution of archaeal viruses of the class *Caudoviricetes*. *PLOS Biol.* **19**, e3001442 (2021). 34752450 10.1371/journal.pbio.3001442 PMC8651126

[38] Baricz, A. et al. Spatio-temporal insights into microbiology of the freshwater-to-hypersaline, oxic-hypoxic-euxinic waters of Ursu Lake. *Environ. Microbiol.* **23**, 3523–3540 (2021). 31894632 10.1111/1462-2920.14909

[39] Shurigin, V. et al. A glimpse of the prokaryotic diversity of the Large Aral Sea reveals novel extremophilic bacterial and archaeal groups. *MicrobiologyOpen* **8**, e00850 (2019). 31058468 10.1002/mbo3.850 PMC6741134

[40] Chernyh, N. A. et al. At the shores of a vanishing sea: microbial communities of Aral and Southern Aral Sea region. *Microbiology* **93**, 1–13 (2024). 10.1134/S0026261723602944

[41] Kheiri, R. et al. Hypersaline Lake Urmia: a potential hotspot for microbial genomic variation. *Sci. Rep.* **13**, 374 (2023). 36611086 10.1038/s41598-023-27429-2 PMC9825399

[42] McGonigle, J. M., Bernau, J. A., Bowen, B. B. & Brazelton, W. J. Robust archaeal and bacterial communities inhabit shallow subsurface sediments of the Bonneville Salt Flats. *mSphere* **4**, e00378–19 (2019). 31462415 10.1128/mSphere.00378-19 PMC6714890

[43] Guglielmin, M. et al. A possible unique ecosystem in the endoglacial hypersaline brines in Antarctica. *Sci. Rep.* **13**, 177 (2023). 36604573 10.1038/s41598-022-27219-2 PMC9814585

[44] Merlino, G., Barozzi, A., Michoud, G., Ngugi, D. K. & Daffonchio, D. Microbial ecology of deep-sea hypersaline anoxic basins. *FEMS Microbiol. Ecol* **94**, fiy085 (2018). 10.1093/femsec/fiy085 29905791

[45] Varrella, S., Tangherlini, M. & Corinaldesi, C. Deep hypersaline anoxic basins as untapped reservoir of polyextremophilic prokaryotes of biotechnological interest. *Mar. Drugs* **18**, 91 (2020). 32019162 10.3390/md18020091 PMC7074082

[46] Nigro, L. M., Elling, F. J., Hinrichs, K. U., Joye, S. B. & Teske, A. Microbial ecology and biogeochemistry of hypersaline sediments in Orca Basin. *PLoS one* **15**, e0231676 (2020). 32315331 10.1371/journal.pone.0231676 PMC7173876

[47] Michoud, G. et al. Fine-scale metabolic discontinuity in a stratified prokaryote microbiome of a Red Sea deep halocline. *ISME J.* **15**, 2351–2365 (2021). 33649556 10.1038/s41396-021-00931-z PMC8319295

[48] Elbehery, A. H. A., Beason, E. & Siam, R. Metagenomic profiling of antibiotic resistance genes in Red Sea brine pools. *Arch. Microbiol.* **205**, 195 (2023). 37061654 10.1007/s00203-023-03531-x

[49] Fisher, L. A. et al. Current state of athalassohaline deep-sea hypersaline anoxic basin research-recommendations for future work and relevance to astrobiology. *Environ. Microbiol.* **23**, 3360–3369 (2021). 33538392 10.1111/1462-2920.15414

[50] Steinle, L. et al. Life on the edge: active microbial communities in the Kryos MgCl_2_-brine basin at very low water activity. *ISME J.* **12**, 1414–1426 (2018). 29666446 10.1038/s41396-018-0107-z PMC5956074

[51] Hallsworth, J. E. et al. Limits of life in MgCl_2_-containing environments: chaotropicity defines the window. *Environ. Microbiol.* **9**, 801–813 (2007). 17298378 10.1111/j.1462-2920.2006.01212.x

[52] Klempay, B. et al. Microbial diversity and activity in Southern California salterns and bitterns: analogues for remnant ocean worlds. *Environ. Microbiol.* **23**, 3825–3839 (2021). 33621409 10.1111/1462-2920.15440

[53] Wilks, J. M., Chen, F., Clark, B. C. & Schneegurt, M. A. Bacterial growth in saturated and eutectic solutions of magnesium sulphate and potassium chlorate with relevance to Mars and the ocean worlds. *Int. J. Astrobiol.* **18**, 502–509 (2019). 33776587 10.1017/S1473550418000502 PMC7992186

[54] Bremer, E. & Krämer, R. Responses of microorganisms to osmotic stress. *Annu. Rev. Microbiol.* **73**, 313–334 (2019). 31180805 10.1146/annurev-micro-020518-115504

[55] Paris, E. R. et al. Single-cell analysis in hypersaline brines predicts a water-activity limit of microbial anabolic activity. *Sci. Adv* **9**, eadj3594 (2023). 38134283 10.1126/sciadv.adj3594 PMC10745694

[56] Saxton, M. A. et al. Sulfate reduction and methanogenesis in the hypersaline deep waters and sediments of a perennially ice-covered lake. *Limnol. Oceanogr.* **66**, 1804–1818 (2021). 10.1002/lno.11723

[57] Rubin, S. S. et al. Prokaryotic diversity and community composition in the Salar de Uyuni, a large scale, chaotropic salt flat. *Environ. Microbiol.* **19**, 3745–3754 (2017). 28752915 10.1111/1462-2920.13876

[58] Martínez, J. M., Escudero, C., Rodríguez, N., Rubin, S. & Amils, R. Subsurface and surface halophile communities of the chaotropic Salar de Uyuni. *Environ. Microbiol.* **23**, 3987–4001 (2021). 33511754 10.1111/1462-2920.15411

[59] Martínez, F. L., Orce, I. G., Rajal, V. B. & Irazusta, V. P. Salar del Hombre Muerto, source of lithium-tolerant bacteria. *Environ. Geochem. Health* **41**, 529–543 (2019). 29995192 10.1007/s10653-018-0148-2

[60] Cubillos, C. F. et al. Insights into the microbiology of the chaotropic brines of Salar de Atacama, Chile. *Front. Microbiol.* **10**, 1611 (2019). 31354691 10.3389/fmicb.2019.01611 PMC6637823

[61] Ordoñez, O. F., Rasuk, M. C., Soria, M. N., Contreras, M. & Farias, M. E. Haloarchaea from the Andean Puna: biological role in the energy metabolism of arsenic. *Microb. Ecol.* **76**, 695–705 (2018). 29520450 10.1007/s00248-018-1159-3

[62] Veloso, M. et al. Diversity, taxonomic novelty, and encoded functions of Salar de Ascotán microbiota, as revealed by metagenome-assembled genomes. *Microorganisms* **11**, 2819 (2023). 38004830 10.3390/microorganisms11112819 PMC10673233

[63] Cavalazzi, B. et al. The Dallol geothermal area, northern Afar (Ethiopia)-an exceptional planetary field analog on earth. *Astrobiology* **19**, 553–578 (2019). 30653331 10.1089/ast.2018.1926 PMC6459281

[64] Belilla, J. et al. Hyperdiverse archaea near life limits at the polyextreme geothermal Dallol area. *Nat. Ecol. Evol.* **3**, 1552–1561 (2019). 31666740 10.1038/s41559-019-1005-0 PMC6837875

[65] Belilla, J. et al. Archaeal overdominance close to life-limiting conditions in geothermally influenced hypersaline lakes at the Danakil Depression, Ethiopia. *Environ. Microbiol.* **23**, 7168–7182 (2021). 34519149 10.1111/1462-2920.15771

[66] Belilla, J. et al. Active microbial airborne dispersal and biomorphs as confounding factors for life detection in the cell-degrading brines of the polyextreme Dallol geothermal field. *mBio* **13**, e00307–e00322 (2022). 35384698 10.1128/mbio.00307-22 PMC9040726

[67] Baas Becking, L. G. M. *Geobiologie of inleiding tot de milieukunde*. W.P. Van Stockum & Zoon, Den Haag (1934).

[68] Brito-Echeverria, J. et al. Occurrence of *Halococcus* spp. in the nostril glands of the seabird *Calonectris diomeda*. *Extremophiles* **13**, 557–565 (2009). 19363644 10.1007/s00792-009-0238-2

[69] Yim, K. J. et al. Occurrence of viable, red-pigmented haloarchaea in the plumage of captive flamingoes. *Sci. Rep.* **5**, 16425 (2015). 26553382 10.1038/srep16425 PMC4639753

[70] Kemp, B. L. et al. The biogeography of Great Salt Lake halophilic archaea: testing the hypothesis of avian mechanical carriers. *Diversity* **10**, 124 (2018). 10.3390/d10040124

[71] Clark, D. R. et al. Biogeography at the limits of life: do extremophilic microbial communities show biogeographical regionalization? *Global Ecol. Biogeogr.* **26**, 1435–1446 (2017). 10.1111/geb.12670

[72] Mora-Ruiz, M. et al. Biogeographical patterns of bacterial and archaeal communities from distant hypersaline environments. *Syst. Appl. Microbiol.* **41**, 139–150 (2018). 29352612 10.1016/j.syapm.2017.10.006

[73] Chen, L. et al. Genomic analyses reveal a low-temperature adapted clade in *Halorubrum*, a widespread haloarchaeon across global hypersaline environments. *BMC Genom.* **24**, 508 (2023). 10.1186/s12864-023-09597-7 PMC10468875 37653415

[74] Tschitschko, B. et al. Genomic variation and biogeography of Antarctic haloarchaea. *Microbiome* **6**, 113 (2018). 29925429 10.1186/s40168-018-0495-3 PMC6011602

[75] Oren, A. Bioenergetic aspects of halophilism. *Microbiol. Mol. Biol. Rev.* **63**, 334–348 (1999). 10357854 10.1128/MMBR.63.2.334-348.1999 PMC98969

[76] Oren, A. Thermodynamic limits to microbial life at high salt concentrations. *Environ. Microbiol.* **13**, 1908–1923 (2011). 21054738 10.1111/j.1462-2920.2010.02365.x

[77] Sorokin, D. Y. et al. *Natronolimnobius sulfurireducens* sp. nov. and *Halalkaliarchaeum desulfuricum* gen. nov., sp. nov., the first sulfur-respiring alkaliphilic haloarchaea from hypersaline alkaline lakes. *Int. J. Syst. Evol. Microbiol.* **69**, 2662–2673 (2019). 31166158 10.1099/ijsem.0.003506

[78] Sorokin, D. Y. et al. Sulfur respiration in a group of facultatively anaerobic natronoarchaea ubiquitous in hypersaline soda lakes. *Front. Microbiol.* **9**, 2359 (2018). 30333814 10.3389/fmicb.2018.02359 PMC6176080

[79] Sorokin, D. Y. et al. Carbohydrate-dependent sulfur respiration in halo(alkali)philic archaea. *Environ. Microbiol.* **23**, 3789–3808 (2021). 33538376 10.1111/1462-2920.15421

[80] Sorokin, D. Y. et al. *Natranaeroarchaeum sulfidigenes* gen. nov., sp. nov., carbohydrate-utilizing sulfur-respiring haloarchaeon from hypersaline soda lakes, a member of a new family *Natronoarchaeaceae* fam. nov. in the order *Halobacteriales*. *Syst. Appl. Microbiol.* **45**, 126356 (2022). 36108543 10.1016/j.syapm.2022.126356

[81] Sorokin, D. Y. et al. *Natranaerofaba carboxydovora* gen. nov., sp. nov., an extremely haloalkaliphilic CO-utilizing acetogen from a hypersaline soda lake representing a novel deep phylogenetic lineage in the class ‘*Natranaerobiia*’. *Environ. Microbiol.* **23**, 3460–3476 (2021). 32955149 10.1111/1462-2920.15241 PMC8359318

[82] King, G. M. Carbon monoxide as a metabolic energy source for extremely halophilic microbes: implications for microbial activity in Mars regolith. *Proc. Natl Acad. Sci. USA* **112**, 4465–4470 (2015). 25831529 10.1073/pnas.1424989112 PMC4394273

[83] Sorokin, D. Y. et al. Anaerobic carboxydotrophy in sulfur-respiring haloarchaea from hypersaline lakes. *ISME J.* **16**, 1534–1546 (2022). 35132120 10.1038/s41396-022-01206-x PMC9123189

[84] Magnuson, E. et al. Active lithoautotrophic and methane-oxidizing microbial community in an anoxic, sub-zero, and hypersaline High Arctic spring. *ISME J.* **16**, 1798–1808 (2022). 35396347 10.1038/s41396-022-01233-8 PMC9213412

[85] Schwab, L. et al. Sulfate reduction and homoacetogenesis at various hypersaline conditions: implications for H_2_ underground gas storage. *Front. Energy Res.* **11**, 1125619 (2023). 10.3389/fenrg.2023.1125619

[86] Medina-Chávez, N. O. et al. Disentangling a metabolic cross-feeding in a halophilic archaea-bacteria consortium. *Front. Microbiol.* **14**, 1276438 (2023). 38179456 10.3389/fmicb.2023.1276438 PMC10764424

[87] Wurtsbaugh, W. A. & Sima, S. Contrasting management and fates of two sister lakes: Great Salt Lake (USA) and Lake Urmia (Iran). *Water* **14**, 3005 (2022). 10.3390/w14193005

[88] Bodaker, I. et al. Comparative community genomics in the Dead Sea: an increasingly extreme environment. *ISME J.* **4**, 399–407 (2010). 20033072 10.1038/ismej.2009.141

[89] Jacob, J. H., Hussein, E. I., Shakhatreh, M. A. K. & Cornelison, C. T. Microbial community analysis of the hypersaline water of the Dead Sea using high-throughput amplicon sequencing. *MicrobiologyOpen* **6**, e00500 (2017). 28677326 10.1002/mbo3.500 PMC5635157

[90] Oren, A. & Garrity, G. M. Valid publication of the names of forty-two phyla of prokaryotes. *Int. J. Syst. Evol. Microbiol.* **71**, 005056 (2021). 10.1099/ijsem.0.005056 34694987

[91] Göker, M. & Oren, A. Valid publication of names of two domains and seven kingdoms of prokaryotes. *Int. J. Syst. Evol. Microbiol.* **74**, 006242 (2024). 10.1099/ijsem.0.006242 38252124

[92] Oren, A. & Göker, M. *Candidatus* list. Lists of names of prokaryotic *Candidatus* phyla. *Int. J. Syst. Evol. Microbiol.* **73**, 005821 (2023). 10.1099/ijsem.0.005821 37159402

